# Analysis of Children's Perception of Triatomine Vectors of Chagas Disease through Drawings: Opportunities for Targeted Health Education

**DOI:** 10.1371/journal.pntd.0003217

**Published:** 2014-10-02

**Authors:** Violetta Yevstigneyeva, Javier Camara-Mejia, Eric Dumonteil

**Affiliations:** 1 Laboratorio de Parasitología, Centro de Investigaciones Regionales “Dr. Hideyo Noguchi”, Universidad Autónoma de Yucatán, Mérida, Yucatán, Mexico; 2 Department of Tropical Medicine, School of Public Health and Tropical Medicine, Tulane University, New Orleans, Louisiana, United States of America; Emory University, United States of America

## Abstract

**Background:**

Chagas disease is a tropical parasitic disease affecting about 10 million people, mostly in the Americas, and transmitted mainly by triatomine bugs. Insect vector control with indoor residual insecticides and the promotion of housing improvement is the main control intervention. The success of such interventions relies on their acceptance and appropriation by communities, which depends on their knowledge and perceptions of both the disease and the vector. In this study, we investigated school-aged children's knowledge and perception on triatomine vectors and Chagas disease to further understand how communities view this vector and the disease in Yucatan, Mexico.

**Methodology/Principal findings:**

We performed an analysis of children's drawings on the theme of triatomines and their house in several rural villages, to explore in an open-ended manner their views, understanding and misconceptions. A total of 261 drawings were collected from children ages 6–12 from four villages. We found that children are very familiar with triatomine vectors, and know very well many aspects of their biology and ecology, and in particular their blood-feeding habits. On the other hand, their drawings suggest that the role of triatomines as vectors of a chronic and severe cardiac disease is less understood, and the main perceived health threat appears limited to the bite itself, as previously observed in adults.

**Conclusions/Significance:**

These results have important implications for the specific design of future education materials and campaigns, and for the promotion of the inclusion of children in raising Chagas disease awareness in these endemic communities.

## Author Summary

Chagas disease is a tropical parasitic disease affecting about 10 million people, mostly in the Americas, and transmitted mainly by triatomine bugs. The elimination of the insect vectors from houses is the main strategy for the prevention of the disease. The success of such interventions relies on their acceptance and appropriation by communities, which depend on their knowledge and perceptions of the disease and the vector. In this study, we performed the first analysis of school-aged children's views of triatomine vectors and Chagas disease in Yucatan, Mexico. We analyzed 261 drawings on the theme of “triatomines and my house” collected in four rural villages. Children appeared very familiar with triatomine vectors, but their role as vectors of a chronic and severe cardiac disease was less understood. These results can help design more specific education materials and campaigns, and promote the inclusion of children in raising Chagas disease awareness in these communities.

## Introduction

Chagas disease is a parasitic disease representing a major public health problem in Latin America, with about 10 million infected people [1]–[3]. In Mexico, there are at least 1–2 million infected people, but the public health importance of the disease is still debated and Chagas disease remains a neglected disease [1], [4]. The disease is caused by the protozoan parasite *Trypanosoma cruzi*, which is transmitted mainly by the feces of hematophagous bugs of the triatomine family, although secondary transmission mechanisms are increasingly contributing to the epidemiology of the disease [5], and it is becoming more urbanized [6]–[9]. After a short acute phase, patients remain chronically infected for many years, in an asymptomatic stage, but 30–40% of them will progress to a chronic chagasic cardiomyopathy, and less frequently to the digestive form of the disease [10].

Control of the disease is mostly based on vector control with intra-domiciliary residual insecticides, sometimes associated with housing improvement, and treatment of infected patients with antiparasitic drugs benznidazole or nifurtimox [5]. A key aspect for the success of these interventions is their acceptance and compliance by the communities and patients, which is in turn affected by their knowledge and perceptions of the disease and the vector [11]–[15]. These socio-cultural aspects of Chagas disease have often been overlooked [16]. Therefore, a more integrative approach is needed as proposed in the ecosystem approach to health (Ecohealth), which promotes the integration of ecological, biological and social aspects for a more effective and sustainable disease control within a context of social and economic development [15], [17], [18]. In a previous study in rural Mayan communities of Yucatan, Mexico, we found that communities had a good knowledge of triatomines and their habits, but most inhabitants had a limited understanding of the transmission mechanisms and clinical manifestations of Chagas disease [15]. Thus, they do not clearly perceive the disease (and its vector) as a serious health threat, in spite of a significant level of house infestation by triatomine vectors (15–54% of houses, [6], [19]–[21]) and a seroprevalence of *T. cruzi* infection of 1–5% in the population [22]–[24]. This lack of threat perception by the communities may negatively affect their participation in, and appropriation of disease control efforts.

On the other hand, children are an understudied population, although they may provide a good representation of community knowledge and perceptions as well. Indeed, Piaget's initial view of children's health knowledge as limited has been challenged by other authors suggesting that they can develop complex mental representations to predict and understand important aspects of their environment [25]. They can also play a key role as health messenger, and thus are an excellent target for health education and the promotion of behavioral changes [26].

A number of different methods have been used to study children's knowledge and beliefs on a particular subject, and drawing analysis has been considered as a powerful tool to analyze children's imagery, as drawings are spontaneous and can reflect children's knowledge (concepts and notions) as well as popular culture and stereotypes [27], [28]. The main advantages of using drawings is that children are used to express themselves graphically in schools, and drawing does not rely on verbal abilities, thus allowing greater freedom of expression by mitigating the limitations and biases which may be present in questionnaires and interviews [29], [30]. Indeed, some concepts and representations can be difficult for a child to express clearly and explicitly in a verbal manner, but may be easily captured in a simple drawing. Thus, one of the main benefits of using drawings as a method to study children's knowledge is that it allows visualization of ideas and concepts in a non-constrained, non-pressured way [31]. Drawing analysis has thus been extensively used to assess children's knowledge and perceptions on a wide range of topics, including the environment, climate change, health education, and illness [30]–[34].

In this study, we investigated school-aged children's knowledge and perception on triatomine vectors and Chagas disease to further understand how communities view this vector and the disease in Yucatan, Mexico. We thus performed an analysis of children's' drawings on the theme of triatomine and their house in several rural villages, aiming to explore how children perceive triatomines, their feeding and resting habits, and their association with Chagas disease. We also evaluated how these views relate to those of adults, as well as to Chagas disease education materials and activities that have been used previously in the studied villages.

## Materials and Methods

### Study participants and design

The study was carried out in the rural villages of Bokoba, Teya, Sudzal and Sanahcat, which are located about 15 to 20 km apart in the central part of the Yucatan state, Mexico. The four villages have very similar housing and socioeconomic conditions, as well as triatomine infestation levels [18], and they have been part of a pilot project on integrated vector control for Chagas disease over the past few years [18], [35]. As such, triatomine collection activities [18], [36], as well as serological surveys [24], and some community education and awareness activities have been taking place in these villages, although the later only focused on adults [15]. Adults were found to be rather knowledgeable on triatomines, but did not associate them well with Chagas disease [15].

Children's drawings were collected through a drawing contest [37], which was conducted in the four study villages between April and December of 2012. Primary school students' aged 6–12 years old from each primary school (six schools in total) from all four villages were invited to participate in the contest. Variables collected included basic demographic data (gender, age, village, name of school and grade).

Formal instructions describing the contest and its requirements were presented in a pamphlet and a poster to the school officials and teachers. Students were asked to use a 50×65 cm poster size sheet of paper of any color for their drawings. Each student was given two weeks to create a drawing on the topic of “My house and triatomines” (“Mi casa y el pic”, as triatomines are most commonly referred to as a “pic” in Mayan). Children were asked to produce the drawings in their own homes and bring them back to school. The following details were requested in the drawings: 1) To present representative aspects of their community, 2) To include triatomine bugs or “pic” (local Mayan name for triatomines) in their drawing, 3) To show where bugs hide inside/around the house, and 4) To show what/whom the bugs feed on (see supplementary Text S1). No further instructions were given, so that children could freely associate triatomines with any situation or characteristics, including or not Chagas disease. We conducted a content analysis of the drawings in order to examine the extent and context of children's knowledge about triatomine vectors and Chagas disease and to understand how previous vector control related activities in the villages have informed their knowledge and understanding. An award ceremony with exposition of the drawings was held in each village after completion of the study, to acknowledge the most informative and creative drawings. This event was used as a Chagas disease awareness event, which included local government officials, school teachers, local health providers and the research team.

### Drawing analysis

Drawings were used as an open-ended approach to explore children's own views and perspectives on triatomines and Chagas disease. Taking the social cognitive learning theory (SCLT) into consideration [38], the aim of the analysis was to capture the diversity of the information communicated through drawings. This theory takes into account the relationship between individual factors, environmental influences, and observational learning and how they collectively influence knowledge and behavior [38]. It makes emphasis on social influence and on how external and internal social reinforcement influence learning and specific behaviors. Thus, children's ability to identify triatomines and depict their knowledge in a drawing format was considered heavily based on the social and environmental conditions that have shaped their perception and understanding.

Because some drawings also included text messages or dialogues, both the image content of the drawings, as well as the text were analyzed. A total of 261 drawings were collected and given an ID number. The drawings were associated with basic demographic information of its author. Children's ages were divided into categories corresponding to 6–7, 8–9 and 10–12 years old.

To analyze the content of the drawings, a qualitative scoring tool was developed. General thematic categories were deductively defined based on established attributes of triatomines and Chagas disease of interest. These themes included triatomine description and activity, resting/hiding locations inside and outside the house, animal and human hosts, signs of disease/suffering and pathology descriptions. A number of inductively developed subthemes/codes emerged from a general review of the drawings. As the result, a total of 61 inductively and deductively defined codes were considered within the general themes. Each drawing was then scored by four independent scorers for the presence or absence of each of the 61 codes. The individual scoring results were combined to create consensus for each theme and subtheme. In case of major discrepancies among scorers, drawings were re-examined collectively, to reach a consensus score. Scores were then used to calculate the frequencies of the presence/absence of the different themes and subthemes in the drawings, and identify the most commonly represented features, as well as some unique features from some drawings. A Chi square statistical test was used to compare frequencies among age groups, gender, and villages using JMP 9 software.

The text written on each drawing was transcribed into an excel sheet and then transferred to Nvivo 7 software for qualitative analysis to identify the most commonly used words as well as define the main themes. The messages were also analyzed to determine the potential source of information for the written text, as well as the type of messages written.

### Ethical statement

The study was approved by both the World Health Organization and the Autonomous University of Yucatan institutional bioethics committees. Parents of the children provided written informed consent to participate in the study.

## Results

**Figure 1:**
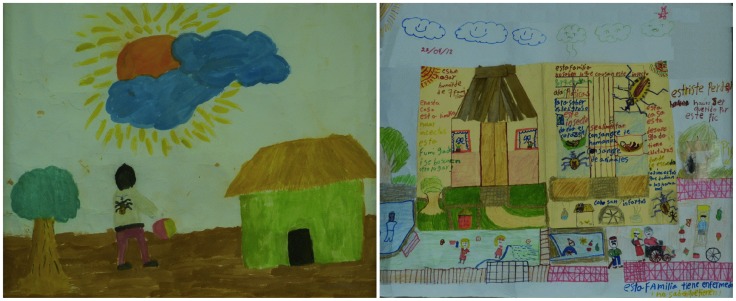
Examples of drawings.

A total of 261 drawings were collected, 92 from Bokoba, 86 from Sanahcat, 45 from Sudzal and 38 from Teya. Of these, 162 were from girls and 99 from boys. There were no significant differences in the content of drawings from boys and girls, although girls tended to draw more triatomines in adult stage of development, while boys tended to draw more animals. The largest number of drawings was from the 10–12 years old age group (112 drawings), followed by the 8–9 years old group (88 drawings), and the 6–7 years old group (45 drawings). No major differences were seen in the contents of the drawings from the different age groups, but as expected the younger age group lacked detail compared to the older age groups. Drawings varied widely in their complexity, but most represented a rather typical house, and the yard surrounding it, with some vegetation and domestic animals. Domestic scenes of playing, sleeping or cooking were often depicted. Triatomine bugs were present both inside and outside the houses. Some drawings presented a very simple scene, while others contained very elaborate health messages (Figure 1 and Supplementary Figure S1).

### Triatomine depiction

**Figure 2:**
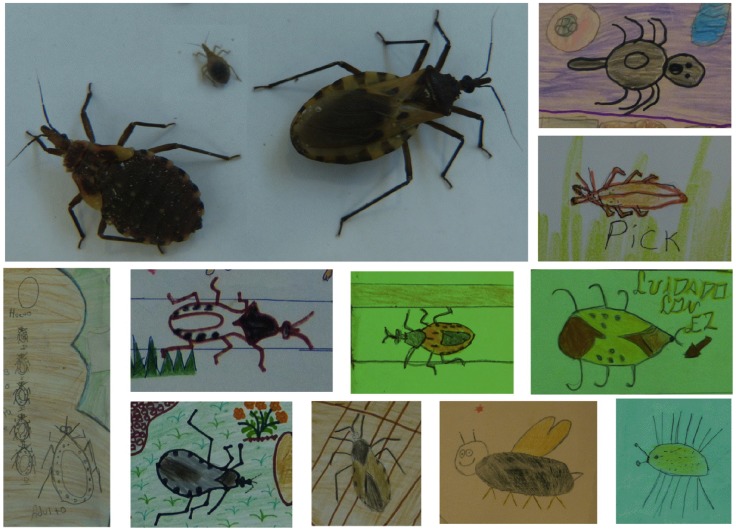
Examples of tratomine descriptions. The photograph shows *Triatoma dimidiata* from the region as adult male (right) and a 4^th^ and 2^nd^ stage nymphs (left). Cropped details of bugs from children's drawing ranged from detailed and accurate representations to indistinguishable bugs, some of which included nymphal stages.

Each drawing had an average of 3.8 bugs drawn, and triatomine bugs could be clearly identified in 93% (243/261) of all drawings (Figure 2). In the large majority of drawings (71%, 186/261), the adult stage of bugs was depicted, while in a minority of drawings nymphal stages were represented (7%, 19/261). The majority of the triatomines in the drawings were scored as having medium detail (34%, 89/261), but a large proportion of drawings also had triatomines presented with very precise details (22%, 58/261), and 30% (78/261) were more difficult to identify (Figure 2). The 6–7 year old group had the largest percentage (40%) of bugs drawn that could not be identified as triatomines (Figure 2). Nonetheless, many of the bugs that could not be readily identified as triatomines were depicted as blood feeding bugs. Thus, the overall quality of the triatomine drawings clearly indicated an excellent recognition and identification of the bugs by the large majority of children. A relatively small proportion (8%, 21/261) of the drawings depicted a night scene, corresponding to the nocturnal behavior of the triatomines, while the majority illustrated a scene in daylight (54%, 140/261), and others were indistinguishable (38%, 99/261).

### Triatomine habitat

**Figure 3:**
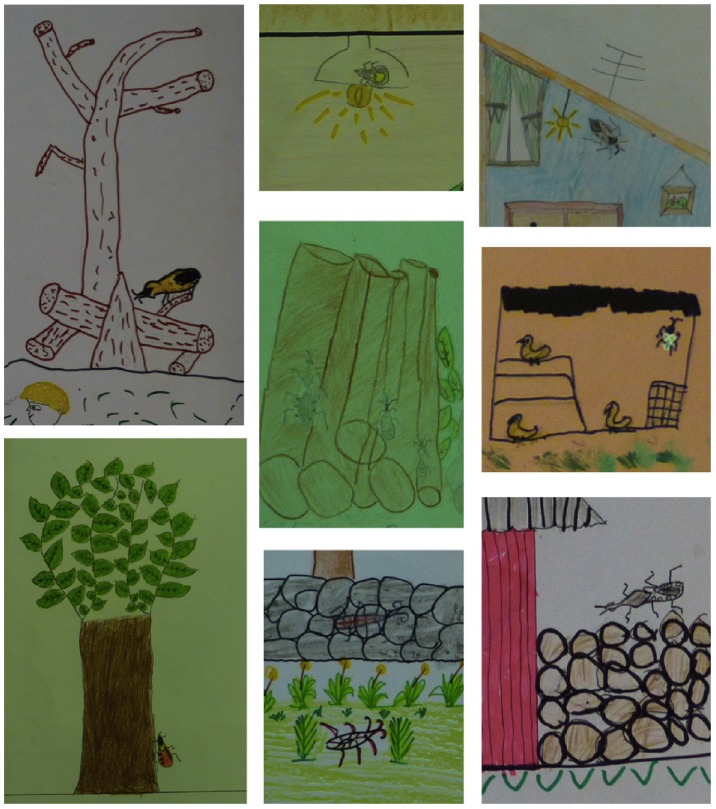
Examples of triatomine habitats. Triatomines were frequently depicted in trees, firewood, grass and yard fence walls made of piled rocks. They were also depicted in chicken coops and other animal corrals, as well as next to lights in a few cases.

Analysis of the location of the bugs, both inside and outside the house, provided information on the perceived habitat and/or resting places of triatomines. The bugs were drawn outside of the house in 81% (212/261) of all drawings and inside in 32% (83/261). The three most common areas outside the house where the bugs were located included trees (36%, 93/261), in the grass (34%, 89/261) and on house walls (27%, 70/261). Bugs were also shown on the fence walls of the yard (made of piled rocks), firewood and/or dead trunks, as well as in chicken coops and animal corrals (Figure 3), which have all been described as potential refuges/habitats for bugs [39].

Inside the house, the most common area where the triatomine was drawn was in the bedroom and more precisely by the bed/hammock in 26% (68/261) of drawings, followed by the floor in 21% (54/261) of drawings. Triatomines were represented next to a light bulb in very few instances (1.5%, 4/261)(Figure 3). Most houses appeared to be made of concrete/cement (63%, 166/261). Many drawings represented triatomines by a door or window of the house (29%, 76/261), in agreement with the flying and invasive behavior of bugs in the region [15], [18], [40].

Some children associated triatomines with trash or junk piles in the yard, and others with water sources and puddles, suggesting some possible confusion with mosquitoes breeding sites [41].

### Triatomine feeding hosts

**Figure 4:**
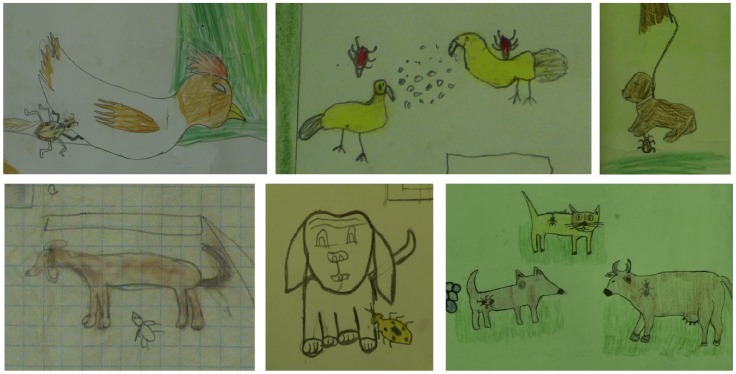
Animal hosts of triatomines. Dogs and chicken were described as the most frequent feeding sources, although feeding was also depicted for a variety of other domestic animals, including cows, horses, cats, sheep or goat.

In only 2% (6/261) of instances triatomines were portrayed flying, and most of the time appeared resting/hiding/feeding. In decreasing frequency, dogs, chicken, horses, pigs, cows and cats were depicted in the yards around the houses, with dogs and chicken also found inside the house in a few drawings (1%, 3/261). Dogs (17%, 44/261) and chicken (13%, 33/261) were the most frequent domestic animals present. Triatomine bugs were clearly blood feeding on animal hosts in 15% (38/261) of the drawings, and portrayed next to or on the animal hosts in an additional 27% (72/261) of the drawings (Figure 4). This clearly illustrated the hematophagous behavior of triatomines. The most frequent animal feeding hosts for bugs were dogs (11/261, 4%), followed by cows (3%, 9/261) and chickens (3%, 8/261).

**Figure 5:**
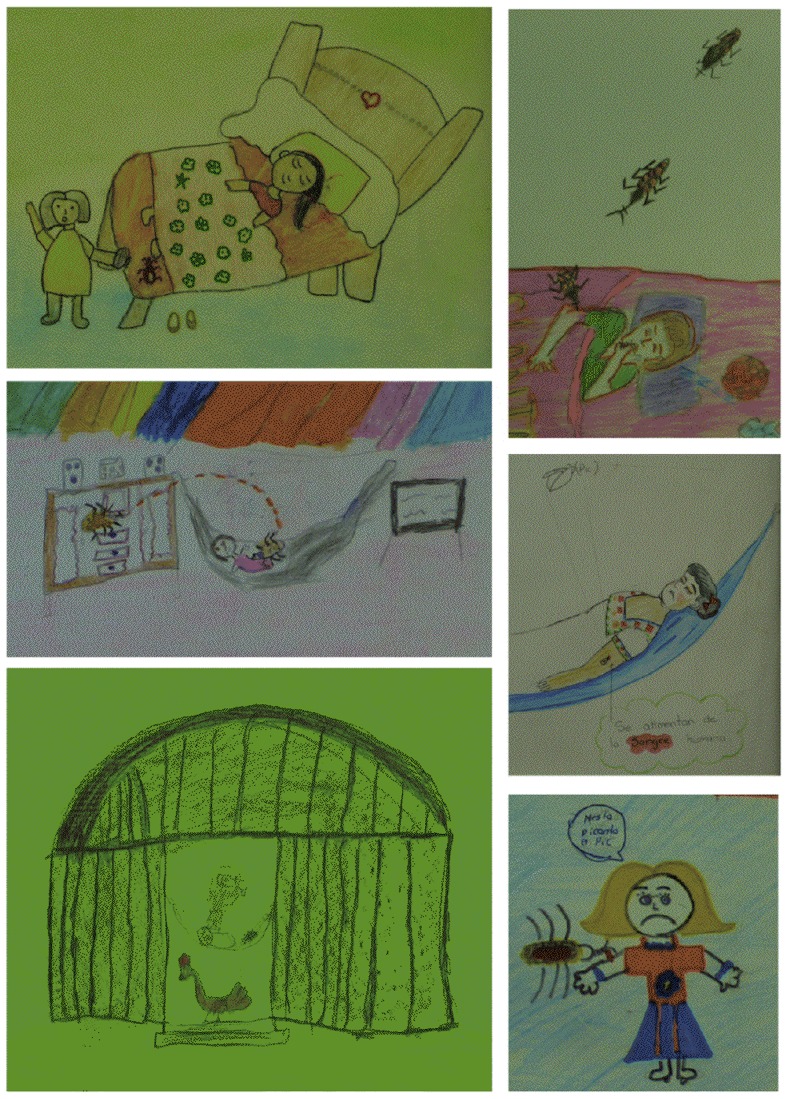
Humans as a feeding source for triatomines. Many children described triatomines feeding on humans, very often while sleeping or resting in their bed or hammock. Triatomines were occasionally depicted flying toward a sleeping person to feed.

Humans were present in 65% of drawings (170/261), and mostly corresponded to children (32%, 84/261). Importantly, triatomines were also described feeding on humans in many drawings (18%, 47/261), and right next to humans in another 38% of drawings (98/261), emphasizing an anthropophylic feeding habit of the bugs (Figure 5). Twenty six percent (68/261) of all drawings showed a human in bed or hammock and 15% (40/261) of all drawings depicted a sleeping person.

A couple of drawings showed a triatomine feeding on flower nectar, and others associated them with water puddles.

### Chagas disease description

**Figure 6:**
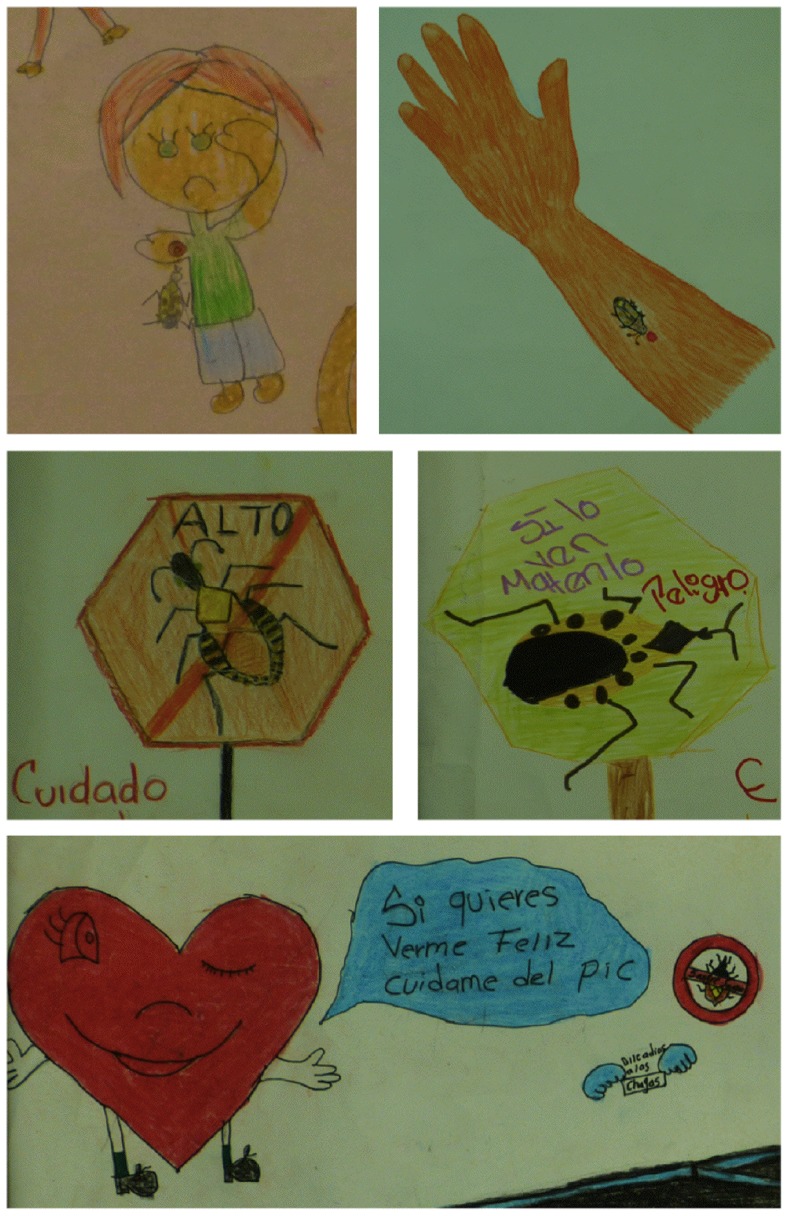
Perceptions of Chagas disease. The most frequently represented threat posed by triatomines was the bite wound, which was frequently depicted in the drawings. Some subjects also appeared to be sad or crying while been bitten. Triatomines were often depicted with warning sings and texts saying “Stop”, “Be carefull”, “If you see it, kill it”. However, triatomines were rarely associated with a chronic disease, and even less frequently with a cardiac disease. The happy heart says “If you want to see me happy, keep me away from the *pic*”.

As an indication of a potential relationship between triatomines and a disease, facial and body expression of the humans were observed. Although in over half of the drawings the facial expression could not be determined, the humans were drawn with a positive, happy expression (smiling, cheering) in 29% of all drawings. In 13% of the drawings the human was visibly discontent and/or in pain and depicted frowning or crying (Figure 5 and 6). Animals were sometimes depicted as scared or in pain as well, when bugs were near them or feeding on them. The most frequent signs of disease depicted were bug bites in 17 drawings (7%), and rash in 10 drawings (4%)(Figure 6). A sick person was present in 2 drawings. Cardiac disease was only rarely depicted (Figure 6). Some children also drew a health center or a doctor.

### Textual messages

Although no specific instructions were provided to the children regarding the inclusion of text in their drawings, over half of them included some textual message or dialogue. Theme analysis indicated that these texts referred to the location of triatomine bugs, their host feeding, general prevention and danger messages, and the association with (Chagas) disease. The most commonly occurring themes were those discussing the location of triatomines and their feeding on humans, and to a lesser extent on animals, in agreement with what was depicted in the drawings.

- Boy 8 year old: “The *pic* lives under rocks and inside old trunks, the *pic* feeds on blood from animals and sometimes from human blood”.
- Boy 11 years old: “The *pic* feeds on animals, they live in the corner of the corral and under rocks.”

Warnings and danger texts were also frequent, labeling triatomines as “Bad”, “Be careful” or “Dangerous”. Triatomine bites were often mentioned and in several instances associated with a severe or fatal disease, although the exact nature of the disease was rarely specified.

- Girl, 12 years old, dialogue between two friends: “– Hey, be careful, there's a bug next to you, don't let it bite you”. – “Thanks, I'm glad you warned me because this is a dangerous bug this one!”
- Girl 11 years old, a bug speaking: “Prepare yourself to be sucked by me as I can be next to you and you can't see me, I take care of your delicious blood, look how fat I am while I take your blood away”.
- Boy 11 years old: “The *pic* feeds on human blood and spreads a fatal disease. With time it develops in human beings”.
- Girl, 12 years old: “The *pic* can be found in dirty houses and a bite can kill you”.
- Girl, 9 years old: “The *pic* bites you and extracts all your strength and gives you a high fever”.

In only a few cases, Chagas disease was mentioned, and in others, reference was made to heart disease. Some text messages were identical among several drawings, suggesting a collective activity. Other messages (8%, 22/261 drawings) were directly copied from education materials distributed by our research group to inform adult participants of our vector control intervention on Chagas disease, and less frequently from materials provided by the Ministry of Health. These suggested some external inputs from a variety of sources in the drawings, either from parents at home or from the teachers in schools, rather than the children's own perceptions. Other routes of transmission were also mentioned a few times including transfusional and congenital. Surprisingly, oral transmission through the consumption of raw or insufficiently cooked meat from infected animals was mentioned in a few drawings.

- Girl 11 years old: “The *pic* is an insect that bites all kinds of animals and persons and causes heart diseases. This little animal feeds on blood. Take care of you children so that they do not get sick. The *pic* can make you seriously sick”.
- Boy 8 year old: “The venom from the *pic* doesn't work fast in the blood of human beings, it can live 10 or 15 years. The venom from the *pic* causes heart failure in human beings”.

### Differences among villages

Because different Chagas disease-related activities had been occurring in each village, we also assessed potential differences in drawings among villages. Indeed, while there had been bug collections in all four villages, pilot vector control interventions had been only performed in Bokoba, Teya and Sudzal, and seroprevalence surveys of the inhabitants had been only performed in Teya and Sudzal.

**Figure 7:**
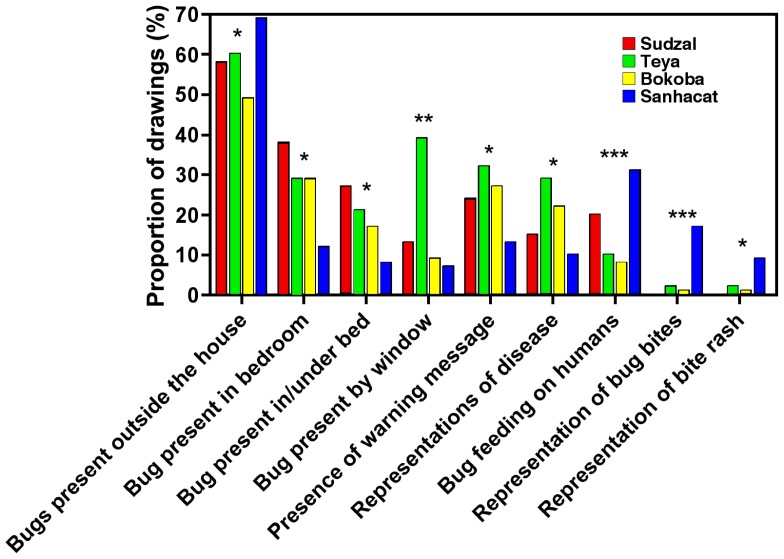
Comparison among drawings from the different villages. The percentage of drawings with the indicated specific features is shown for each village. *, ** and *** indicate significant differences among the four villages (χ^2^ tests, *P*<0.05, *P*<0.01, and *P*<0.001, respectively).

Overall, drawings from the village of Sanahcat had the highest proportion of drawings without triatomines clearly represented (**X** ^2^ = 19.3, *P* = 0.022). Bugs were also less frequently depicted inside the house (**X** ^2^ = 18.9, *P* = 0.026), as well as in a bedroom (**X** ^2^ = 16.3, *P* = 0.012) and on/under a bed (**X** ^2^ = 8.7, *P* = 0.033; Figure 7). This suggested that in this village, bugs are more perceived as sylvatic/peridomestic, and not frequently entering houses. Also, drawings from Sanahcat presented significantly fewer representations of warning and danger images (**X** ^2^ = 9.1, *P* = 0.027), as well as fewer references to disease (**X** ^2^ = 8.4, *P* = 0.038; Figure 7). Associations of triatomines with water sources and puddles were only seen in drawings from the villages of Sanahcat and Sudzal. This suggested a lesser familiarity with triatomines of the children from Sanahcat.

Nonetheless, in the villages of Sanahcat and Sudzal, bugs were more frequently depicted as blood feeding on humans compared to the other villages (**X** ^2^ = 35.5, *P*<0.0001). In Sanahcat, there were more associations of the bugs with bites (**X** ^2^ = 25.5, *P*<0.0001) and rash (**X** ^2^ = 11.1, *P* = 0.011) on human subjects, which were more rarely depicted in drawings from the other villages (Figure 7), suggesting a greater perception of triatomines as a health threat in Sanahcat.

There were also some differences in the perceived habitats. Bugs were more frequently described in association with chicken coops and animal corrals in Sudzal (**X** ^2^ = 55.2, *P*<0.0001), although this may be a simple reflection of the overall higher abundance of domestic animals in the drawings from this village. In Sudzal and Teya, bugs were also more frequently located on yard fences compared to the other villages (**X** ^2^ = 21.6, *P*<0.001). Interestingly, in Teya bugs were much more frequently depicted near windows and doors (**X** ^2^ = 15.4, *P* = 0.0015 and **X** ^2^ = 9.5, *P* = 0.023; Figure 7), which seems clearly related to the pilot installation of window screens performed previously [35]. However, the other differences among drawings from the different villages could not be clearly associated with the nature of previous Chagas disease-related activities.

## Discussion

The knowledge and perceptions of communities related to insect vectors and the disease they transmit are crucial aspects which influence community acceptance of, and participation in, vector and disease control activities [15], [17]. Indeed, communities need to appropriate the interventions for these to be self-sustained and effective. In that respect, children have been given little attention, even though they may provide a good representation of community knowledge and perceptions. They can also play the role of health messengers, and thus are an excellent target for health education and the promotion of behavior changes [26], which is subsequently spread to other family members and friends. To our knowledge, our study is the first to focus on children's views of triatomine vectors and Chagas disease based on drawing analysis.

A first striking observation was the large amount of information presented in the drawings and the attention to detail in many of them. This underlies the breadth and accuracy of children's understanding and knowledge of triatomines and their behavior, as well as indicates that children are very familiar with these bugs. Indeed, the unambiguous precision of many drawings clearly suggested that children have an intimate knowledge of the bugs based on their own observations and experiences. This is in agreement with previous observations of a rather elevated house infestation by triatomines in the region, and further highlights the risk of *T. cruzi* transmission to humans. Indeed, serological surveys have indicated a seroprevalence of 1–5% in the region [22], [23], and cases of children seropositive for *T. cruzi* have also been reported [24]. The greater familiarity with adult bugs compared to nymphal stages may also be a reflection of house infestation being mostly caused by invasive adult bugs, with limited colonization (defined by the presence of nymphs), as reported previously [19], [21], [40], [42]. Furthermore, several of the bug habitats and hiding places as well as blood feeding sources depicted by children, including firewood, rock piles and walls, artificial lights, chicken coops and dogs, have been identified as important determinants for house infestation by *T. dimidiata* in the region [6], [18], [43]. Thus, as observed in several other studies on children's drawings, analysis of their content is a powerful tool to explore children's views on a variety of issues including health [28], [34], and these studies consistently illustrate that children's knowledge and perceptions are much more extensive than we think or assume. For example, children have been found to be able to represent complex concepts related to AIDS [33] or genetics [25], among other themes [27], [29], [31], [44], [45]. With respect to triatomines, their knowledge appears similar to, or possibly even more detailed than that we observed in adults from the same villages [15].

Nonetheless, a few incorrect depictions were also occasionally observed, such as triatomines breeding/hiding in water sources and puddles, as well as nectar feeding on flowers. These seem to have arisen from confusion with mosquitoes, and may be related to the extensive education and diffusion campaigns on mosquitoes as part of Dengue fever prevention activities. Indeed, current dengue control efforts are based in part on the identification and removal of mosquito breeding containers from backyards and homes, which is promoted by multimedia campaigns [41]. The idea of (Chagas) disease transmission by the consumption of raw/uncooked meat from infected animals is surprising as it seems to be rather rare. Indeed, while several Chagas disease outbreaks caused by the ingestion of foods contaminated by bug feces (mostly fruit juice) have been reported in South America in recent years [46], there are only two suspected cases of infection by consumption of raw meat or blood from infected animals, suggesting that more studies are needed to establish the epidemiologic relevance of this mechanism of transmission in humans.

The blood feeding of triatomines appears also well understood by the large majority of children, with several of the established feeding sources such as dogs and chickens [47]; Dumonteil et al., unpublished data] well represented in their drawings. Nonetheless, as previously observed in adults [15], there is a poor association of triatomines with a severe chronic disease, and with Chagas disease in particular. Most of their preoccupations are associated with the bug bite itself, which may cause pain or skin rash. This may be due to the difficulty in depicting a severe cardiac disease, even though the children appeared to have a high level of imagination and disposition to communicate, as evidenced by the danger images and the addition of creative text warnings in the drawings. Human expressions and emotions may also not be the best indicator of an association of bugs with disease, as the ability to depict and understand emotions is still undergoing development in school-aged children [44], [45], which may explain the large number of drawings with happy faces we observed. Nonetheless, the depiction of a severe disease, and of a cardiac pathology in particular, was rather uncommon. In comparison, in a study on genetics, children sometimes associated genetics with disease [25], while in a drawing study on AIDS, the majority of children clearly depicted bedridden sick persons and death in their representations of disease long-term outcomes [33]. The lack of perception of triatomines as a vector of a severe cardiac disease may also reflect the invisible nature of the disease, of which they only have heard of within the social context of their communities (*i.e.* observational learning as in SCLT [38]), compared to their more vivid experiences with triatomines. Further approaches should help clarify children's perceptions of Chagas disease.

Taken together, these observations pinpoint to several specific aspects of Chagas disease that should be strengthened in future education and diffusion messages. First, it would be of key importance to take advantage of the excellent knowledge that children and adults have of triatomines to further build upon this knowledge in future materials. Second, the disease should be better explained, in terms of its symptoms, clinical manifestations and outcomes, to ensure that the severity and nature of Chagas disease is well understood by communities. Third, its relationship with triatomines, including the mechanisms of parasite transmission via triatomine feces upon blood feeding and the slow development of the disease, needs to be reinforced.

A potential bias of our design was that children were given two weeks to complete their drawings as it was organized as a contest, while in most previous studies on children's drawings they were only given a much shorter time (30 min–2 h) to realize their drawings on site. Their drawings may thus be seen as less spontaneous and more researched, but such contests have been used before with success to understand children's perceptions [37]. The fact that several children copied text messages from Chagas disease awareness materials clearly indicate that they did some research and/or had some potential input from parents or teachers. In any case, this should be seen positively as it demonstrates that these awareness materials are being rather extensively circulated, and more importantly socialized within the entire community, thereby effectively contributing to increased community knowledge and awareness on Chagas disease. However, not all the differences we observed among drawings from the different villages could be attributed to differences in the previous Chagas disease awareness and research-related activities that were performed in these villages, suggesting that there may also be intrinsic differences in communities' knowledge and perceptions of triatomines and Chagas disease.

In conclusion, our exploration of children's knowledge and perceptions of triatomines and Chagas disease through drawings confirmed that this approach is a powerful tool to explore, in an open-ended manner, the views of children, their understanding and misconceptions of health-related topics. We found that children are very familiar with triatomine vectors, and know very well many aspects of their biology and ecology, and in particular their blood-feeding habits. On the other hand, their drawings suggest that the role of triatomines as vectors of a chronic and severe cardiac disease is less understood, and the main perceived health threat appears limited to the bite itself, as previously observed in adults. These results have important implications for the specific design of future education materials and campaigns, and the inclusion of children in promoting Chagas disease awareness in these endemic communities.

## Supporting Information

Checklist S1 STROBE checklist.
(DOC)

Figure S1 Additional examples of drawings.
(TIF)

Text S1 Contest instructions in Spanish.
(DOCX)
