# Supplementary material for: Analysis of Children's Perception of Triatomine Vectors of Chagas Disease through Drawings: Opportunities for Targeted Health Education
Source: PLoS Negl Trop Dis. 2014 Oct 2;8(10):e3217. doi: 10.1371/journal.pntd.0003217 (PMC4183480; doi:10.1371/journal.pntd.0003217)
Supplement: Text S1 — Contest instructions in Spanish. (DOCX) [file pntd.0003217.s003.docx]

**C O N V O C A T O R I A D E D I B U J O**

El CIR “Dr. Hideyo Noguchi” de la UADY, el H. Ayuntamiento de Bokobá, Yucatán y el Centro de Salud convocan a todos los niños de esta comunidad a participar en el **PRIMER CONCURSO DE DIBUJO INFANTIL “MI CASA Y EL PIC”** que se llevará a cabo de acuerdo a las siguientes bases:

1.- Podrán participar todos los NIÑOS y NIÑAS de edad escolar que vivan en la comunidad de Bokobá, Yuc.

2.- CATEGORÍAS: INFANTIL “A” (de 6 a 7 años), INFANTIL “B” (de 8 a 9 años) INFANTIL “C” (de 10 años en adelante).

3.-TAMAÑO DEL DIBUJO: En un pliego de cartulina blanca o color.

4.-PUNTOS A CALIFICAR: -Presentación del dibujo (nombre completo con apellidos, edad, nombre de la escuela y dirección de casa) -Creatividad y colorido -Aspectos representativos de la comunidad –Incluir el insecto o insectos “Pic” en el dibujo -Lugares donde se esconde el Pic en la casa -¿De qué y de quienes se alimenta el Pic?.

5.-FECHA LÍMITE PARA LA ENTREGA DE DIBUJOS: miércoles 26 de septiembre de 2012.

6.-LUGARES DE RECEPCIÓN: En la dirección de tu escuela primaria.

7.-FECHA DE EXPOSICIÓN DE DIBUJOS: miércoles 03 de octubre de 2012 en los corredores del palacio municipal a las 4 de la tarde.

8.-PREMIACIÓN: Premios atractivos para los 3 primeros lugares de cada categoría.
